# Clinical, Pathological, and Prognostic Characteristics of Glomerulonephritis Related to Staphylococcal Infection

**DOI:** 10.1097/MD.0000000000003386

**Published:** 2016-04-18

**Authors:** Si-Yang Wang, Ru Bu, Qi Zhang, Shuang Liang, Jie Wu, Xue-Guang Zhang Shu-Wen Liu, Guang-Yan Cai, Xiang-Mei Chen

**Affiliations:** From the Department of Nephrology, Chinese PLA General Hospital, Chinese PLA Institute of Nephrology, State Key Laboratory of Kidney Diseases, National Clinical Research Center for Kidney Diseases, Beijing, China.

## Abstract

Staphylococcal infection has become a common cause of postinfectious glomerulonephritis in the past 3 decades. Because few investigations focus on this disease, the demographics and clinicopathological features of glomerulonephritis related to staphylococcal infection are not well characterized.

We conducted a pooled analysis of published literature in electronic databases and analyzed the clinical features, laboratory findings, and histopathological changes. The patients were divided into 4 groups based on their prognosis: remission, persistent renal dysfunction, end-stage renal disease (ESRD), or death. A logistic regression model was used to identify the determinants of disease outcome.

A total of 83 (64 men) patients with glomerulonephritis related to staphylococcal infection from 31 reports were analyzed. The mean age was 58 years (58 ± 17). Majority of the reports originated from Taiwan, Japan, and the United States. Clinical characteristics of the cases were hematuria (82/83), proteinuria (78/83), and acute kidney injury (75/83). Visceral abscesses (26/83) and skin infections (24/83) were the common sites of infection. Methicillin-resistant *Staphylococcus aureus* was the most common pathogen. The dominant or codominant deposition of IgA or C3 along the glomeruli was an important feature identified by immunofluorescence. There were 19 patients (22.9%) that progressed to dialysis-dependent ESRD. Twelve patients (14.5%) died. A univariate regression analysis indicated that diabetes mellitus (DM) (odds ratio [OR] 2.96; 95% confidence interval [CI] 1.03–8.48; *P* = 0.04) and age (OR 4.80; 95% CI 1.84–12.53; *P* = 0.001) were risk factors for ESRD or death. A multivariate regression analysis also revealed that age (OR 4.90; 95% CI 1.82–13.18; *P* = 0.002) and DM (OR 3.07; 95% CI 0.98–9.59; *P* = 0.05) were independent risk factors for unfavorable prognosis.

Glomerulonephritis related to staphylococcal infection has different features than typical postinfectious glomerulonephritis. The diagnosis of glomerulonephritis related to staphylococcal infection relies on immunofluorescence and electron microscopy findings. Age and DM are independent risk factors of poor prognosis for glomerulonephritis related to staphylococcal infection.

## INTRODUCTION

The incidence of postinfectious glomerulonephritis (PIGN) has decreased rapidly in developed countries over the past 50 years. There has been an important shift in the epidemiology, bacteriology, and outcomes of this disease due to improvements in living conditions and the early use of effective antibiotics.^1^ The responsible organism is predominantly *Staphylococcus*. Since Koyama et al^2^ reported glomerulonephritis associated with methicillin-resistant *Staphylococcus aureus* (MRSA) infection for the first time, glomerulonephritis related to staphylococcal infection has become recognized as the more severe disease manifestation, which is 3-fold more common in the older patients in developed countries.^3^ Glomerulonephritis related to staphylococcal infection is pathologically characterized by mesangial and/or endocapillary proliferation with or without extracapillary lesions, which is containing IgA-predominant or IgA-codominant glomerular depositions by immunofluorescence (IF). There is also frequently interstitial inflammation observed in the renal histopathology. Glomerulonephritis related to staphylococcal infection is also defined as an IgA-dominant PIGN or *Staphylococcus* infection-associated glomerulonephritis mimicking IgA nephropathy.^4^ Diabetes is a major risk factor for glomerulonephritis related to staphylococcal infection, and is associated with the increased skin and mucosal colonization.^5^ The pathogenesis involves staphylococcal neutral phosphatase,^6^ a 70-kDa protein (p70)^7^ that has affinities for both human IgG and rat glomerular basement membrane. A third potential nephritogenic staphylococcal antigen is staphylokinase (also called “staphylococcal neutral protease III”). This molecule is produced by both aureus and non-aureus strains of staphylococci and is known to activate plasmin.^8^ The pathogenesis of glomerulonephritis related to staphylococcal infection is not fully understood. Bacterial superantigens may play a key role in the pathological change. Koyama et al found that the staphylococcal antigens like enterotoxins C and A and toxic shock syndrome toxin (TSST)-1 act as a superantigen and it can bind directly to the major histocompatibility complex class II molecules on antigen-presenting cells. This complex then binds to the T-cell receptor without major histocompatibility complex restriction. The interaction results in massive T-cell activation and a subsequent cytokine burst. The cytokines cause polyclonal B-cell activation that induces the production of IgA, IgG, and IgM. These components have a natural specificity for staphylococcal antigens such as those in the cell envelope.^2^

There are currently only a small number of published case reports or clinical investigations on glomerulonephritis related to staphylococcal infection. Therefore, this new type of glomerulonephritis is not well characterized in terms of demographics, clinical features, risk factors, and renal biopsy findings. In this study, we conducted a pooled analysis of cases with glomerulonephritis related to staphylococcal infection to improve the recognition of this disease.

## METHODS

### Review Strategy and Literature Search

In our study, ethical approval was not necessary, as this study is a pooled analysis, which is based on the published data. We searched the literature available in electronic databases (PubMed/MEDLINE, EMBASE, Science Citation Index, OVID evidence-based medicine). The key words that were used included “glomerulonephritis,” “Staphylococcus,” and “infection.” The search was confined to human studies and there was no language restriction. The search yielded 58 reports. One study was excluded because the patients had nephropathy combined with infection.^9^ Six references were excluded because of publication type (ie, review).^5,10–14^ Seventeen studies were also excluded due to unavailable original demographic and clinicopathological data.^3,15–30^ Three studies were excluded due to a lack of renal biopsy.^31–33^ Finally, 31 studies concerning glomerulonephritis related to staphylococcal infection were obtained and qualified.^2,4,8,34–61^

### Subject Selection

The following criteria were used to define the glomerulonephritis related to staphylococcal infection in our study: clinical or laboratory evidence of infection preceding or at the onset of glomerulonephritis; glomerulonephritis confirmed by renal biopsy; and *Staphylococcus* isolated from cultures. The following exclusion criteria were used to eliminate unqualified cases: no renal biopsy results and insufficient data (ie, absence of IF pathology findings).

### Outcomes and Measurements

Primary data from the original reports were not uniformly available. Thus, acute kidney injury (AKI) was defined as an increase in serum creatinine of at least 0.5 mg/dL above the baseline level. To conduct the outcome analysis, the patients were divided into the following 4 groups based on their prognosis: remission, persistent renal dysfunction (PRD), end-stage renal disease (ESRD), and death. Remission was defined as normalization of serum creatinine to baseline levels or to a creatinine <1.2 mg/dL (for patients in whom baseline creatinine levels were unavailable). PRD was defined by a 0.2 mg/dL elevation of serum creatinine above baseline levels or follow-up creatinine >1.2 mg/dL (for those in whom baseline levels were unavailable). ESRD was defined as requiring maintenance dialysis therapy.

### Statistical Analysis

The data analysis was performed using SPSS 17.0 software (SPSS Inc., Chicago, IL). The mean ± standard deviation or the median and range were used to report the continuous variables. The univariate and multivariate analyses were performed using logistic regression analysis. Statistical significance was assumed at *P* <0.05.

## RESULTS

### Epidemiology and Clinical Features

The demographic and clinicopathologic characteristics of 83 patients with glomerulonephritis related to staphylococcal infection were summarized in Table 1. The majority of the patients were male (64/83), and the male-to-female ratio is 3.37:1. The largest patient population was Asian (49.4%), and the second largest was American (48.2%). European populations including Spanish, British, and Italian patients were also reported in this analysis. The percentage of adults with PIGN older than 65 years of age increased from 4% to 6%, to 34% in the past 40 years.^26^ This pooled analysis showed that 34 of the 83 patients (41%) were older (over 65 years of age). The patients had an average age of 58 years (58 ± 17, range 13–90 years). Hypocomplementemia was detected in 45 of the 83 patients (54.2%). AKI (75/83) with heavy proteinuria (78/83) and hematuria (82/83) were typical features. Proteinuria was common (3.85 ± 3.44 g/d, range 0.15–15 g/d), and the prevalence of the nephrotic range was 20.8%. The mean baseline serum creatinine level was 2.29 mg/dL (2.29 ± 2.16, range 0.5–9.9 mg/dL), and the mean peak serum creatinine level was 5.67 mg/dL (5.67 ± 3.23 mg/dL, range 1.3–12.29 mg/dL). *S aureus* was the most common causative agent, in which 55 patients (66.3%) were infected with MRSA. Twenty-one patients (25.3%) were infected with methicillin-sensitive *S aureus* (MSSA). The remaining patients tested positive for *Staphylococcus epidermidis* infection (4.8%). The other patients were infected with *S aureus*, but details were unavailable. The site of infection was identified in all of the patients and the locations were diverse. The skin and viscera were the most frequent sites of staphylococcal infection with infection rates of 28.9% and 31.3%, respectively. The types of skin infections reported included cellulitis, ulcers, surgical wound infections, and skin abscesses. The majority of visceral infection sites included pneumonia, endocarditis, and osteomyelitis. A variety of other infections were also prevalent. These infections included joint infections, respiratory infections, and bloodstream infections. There were no patients with a history of renal disease, and glomerulonephritis appeared after the infection in all cases. Concomitant diseases included diabetes mellitus (DM) (20 patients, 24.1%), hypertension (15 patients, 18.1%), heart diseases (18 patients, 21.7%), and carcinoma (14 patients, 16.9%).

**TABLE 1:**
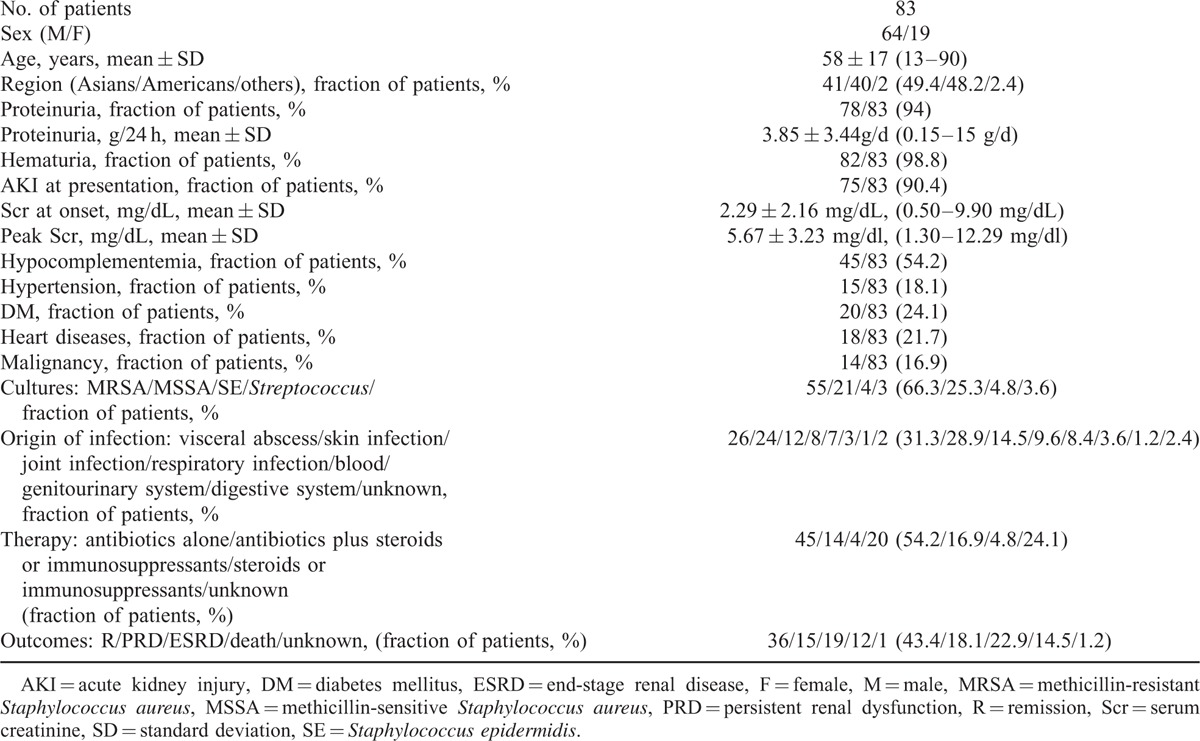
Demographic and Clinical Features of Patients with *Staphylococcus* Infection-associated Glomerulonephritis

### Pathology Findings

There were a variety of renal pathologies including mesangial and/or endocapillary proliferation with varying degrees of crescent formation and tubulointerstitial nephritis. The light microscopy (LM) findings differed based on renal biopsy analysis (Table 2), and ranged from mesangial hypercellularity to diffuse proliferative glomerulonephritis (DPGN). Twenty-four patients had DPGN associated with infiltration of neutrophils and mononucleated cells. Mesangial proliferative glomerulonephritis (MsPGN) was also observed. Nineteen patients had endocapillary proliferation with inflammatory cell infiltration. Necrotizing crescentic glomerulonephritis and cellular crescents were present in 30 patients. Twenty-five patients exhibited acute tubular necrosis (ATN). Tubular atrophy (TA) was found in 22 patients. There was marked underlying diabetic glomerulosclerosis (DGS) found in 13 cases with diabetes.

**TABLE 2:**
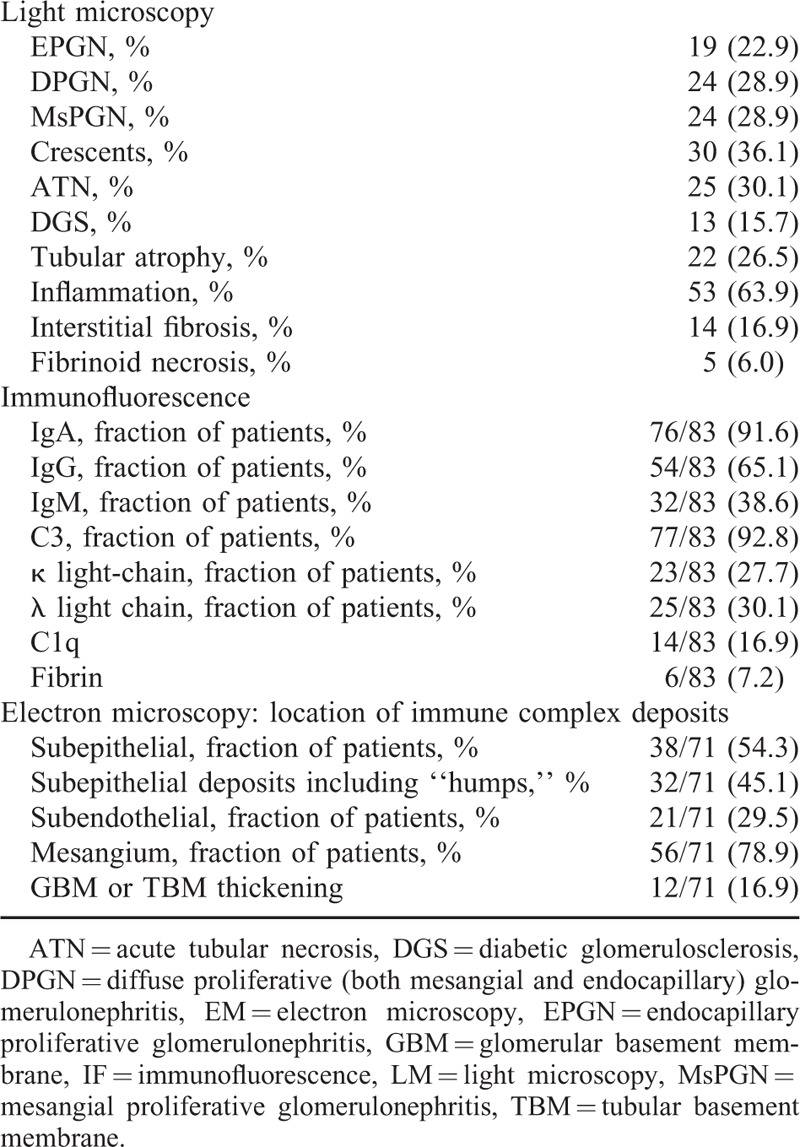
Histopathology Features of Patients with *Staphylococcus* Infection-associated Glomerulonephritis

The most common staining pattern observed by IF was dominant or codominant IgA immune complexes deposited in the glomeruli (76/83). IgA was deposited in the mesangial mesangium in 67 patients, and 29 patients also exhibited IgA deposits on the glomerular capillary wall. C3 was also the dominant or codominant immune reactant detected in the glomeruli of 77 patients. Seventy-three cases presented codeposition of IgA and C3. Fifty-four patients were found IgG deposits in mesangial and/or glomerular capillary wall. There was trace to 3+ IgM staining in the mesangium or glomerular capillary wall in 32 patients. Fourteen patients showed IF staining for C1q and 6 patients showed weak staining for fibrin. Staining for kappa was detected in 23 cases and lambda light chain was present in 25 cases.

An ultrastructural analysis was performed in 70 cases. The electron microscopy (EM) analysis detected mesangial deposits in 56 patients (78.9%) and subendothelial deposits in 21 patients (29.5%). Typical subepithelial “hump-shaped” electron-dense deposits were present in 32 patients (45.1%). Twelve patients (16.9%) displayed variable degrees of thickening of the glomerular and tubular basement membranes.

### Treatment and Outcomes

The use of anti-infective drugs was the typical treatment for most cases. Antibiotic therapy was administered to 59 patients. Fourteen patients also received steroids/immunosuppressants. The remaining patients who had no definite episodes of infection were conservatively treated. There was one case report of a 48 year-old man who failed to respond to antibiotic treatment alone, but responded to a combination of steroids and antibiotics.^44^ Despite the effect of steroids, their use inhibits the immune system and can increase the risk of infection relapse. Thus, the use of steroids for PIGN treatment is still questionable. Patient outcomes were variable, except the outcome of 1 patient which was unavailable. The renal function of 36 patients (43.4%) achieved remission and 15 patients (18.1%) had PRD. Nineteen patients (22.9%) progressed to ESRD and remained dialysis-dependent. Twelve of the total patients (14.5%) died. Approximately, 25.5% of the patients who progressed to ESRD or death had underlying DM compared with 15.7% of the patients who recovered or had PRD (Table 3). This result suggests that an unfavorable outcome may be partly ascribed to DM. Certain histological findings may lead to different outcomes. Adult patients aged >65 years or those with underlying chronic diseases have a more guarded prognosis.

**TABLE 3:**
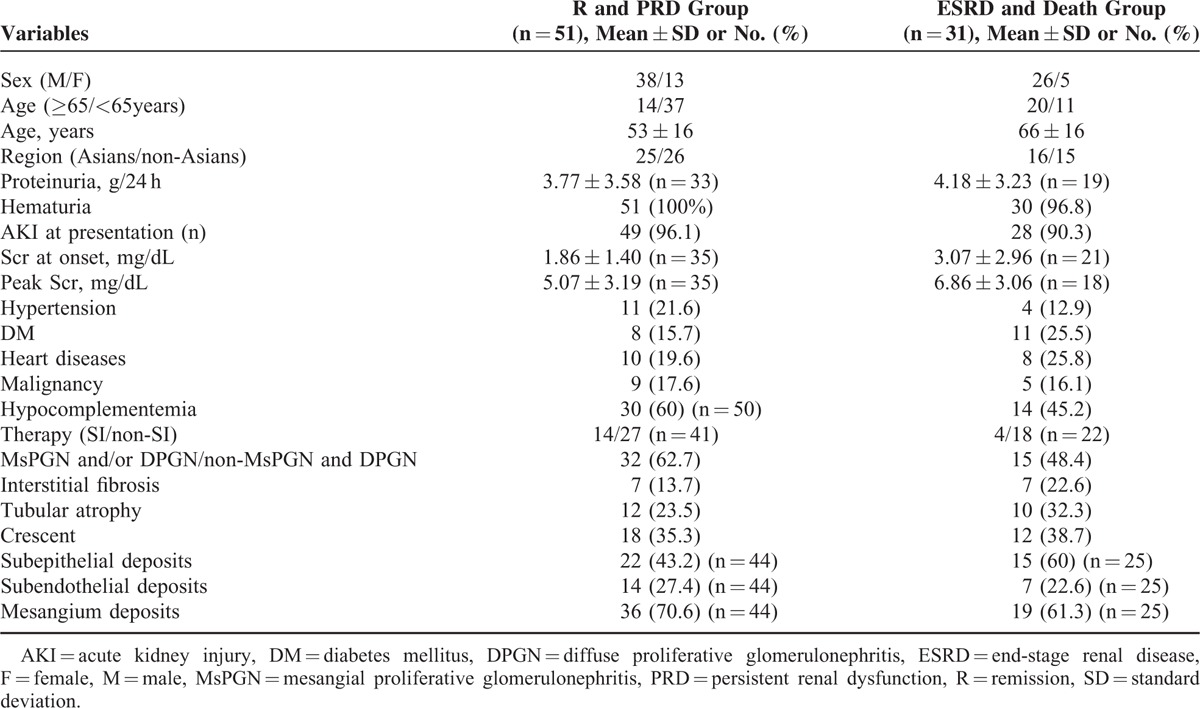
Comparison of Clinical Parameters Between 2 Outcome Groups

### Univariate and Multivariate Analyses

To access the risk factors of prognosis, we separated the patients into 2 groups based on whether they progressed to ESRD or died (Table 3). The risk factors are summarized in Table 4. A univariate logistic regression analysis showed that the correlates for ESRD and death were age (odds ratio [OR] 4.80; 95% confidence interval [CI] 1.84–12.53; *P* = 0.001) and DM (OR 2.96; 95% CI 1.03–8.48; *P* = 0.04). A multivariate logistic regression analysis also revealed that age (OR 4.90; 95% CI 1.82–13.18; *P* = 0.002) and DM (OR 3.07; 95% CI 0.98–9.59; *P* = 0.05) were statistically significant risk factors for ESRD or death.

**TABLE 4:**
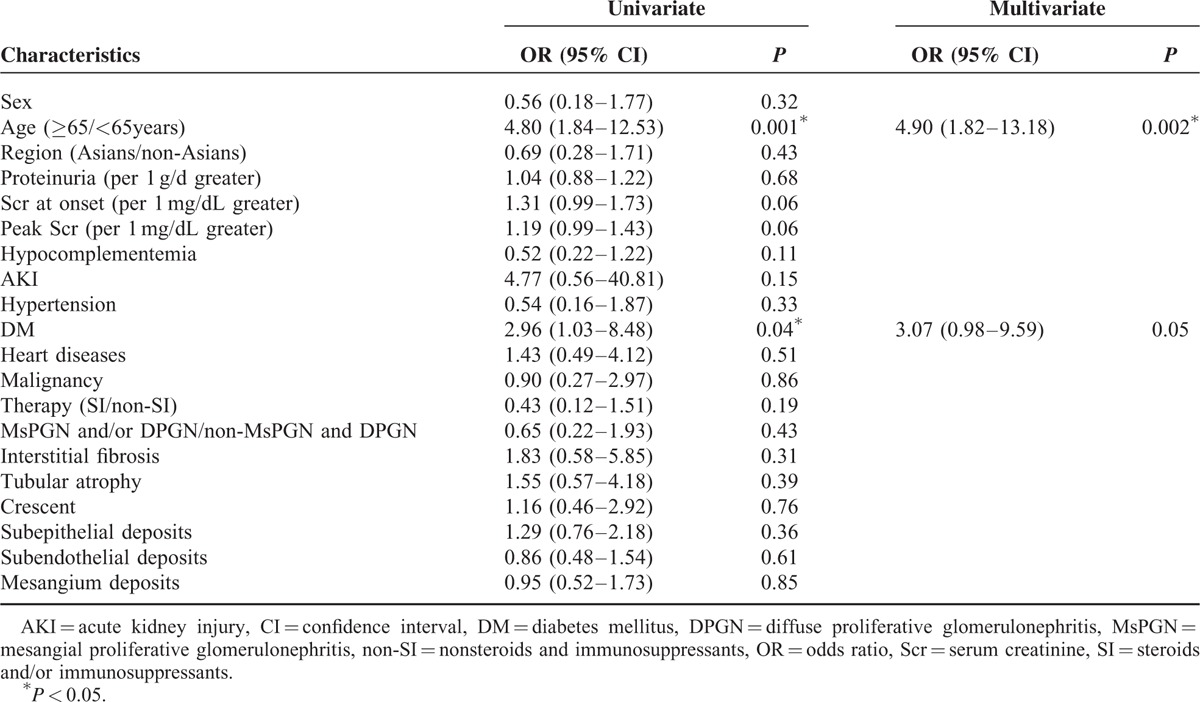
Univariate and Multivariate Logistic Regression Analysis of Factors at Baseline Influencing Unfavorable Prognosis of *Staphylococcus* Infection-associated Glomerulonephritis

## DISCUSSION

*Staphylococcus* has become a common infection in developed countries, and it is 3-fold more common in the older patients.^3^ However, larger-scale clinical datasets are still limited in the available literature. This is the first pooled study of glomerulonephritis related to staphylococcal infection, which summarize the clinical, pathological, and prognostic characteristics of this disorder. The data indicated that glomerulonephritis related to staphylococcal infection appeared more commonly in the older patients who usually presented with AKI, proteinuria, and hematuria. Staphylococcal enterotoxins produced by MRSA-GN act as superantigens^62^ and presumably contribute to the pathogenesis of this form of glomerular nephritis.^2^ A large number of cases are caused by *S aureus*, in which MRSA is more common than MSSA.^26^

Because of the different treatments and prognoses of these diseases, it is important to discriminate between glomerulonephritis related to staphylococcal infection and typical PIGN. The characteristics of patients with glomerulonephritis related to staphylococcal infection were different from patients with PIGN syndrome. The most common light microscopic patterns of PIGN were diffuse (53%), focal (28%), and mesangial (13%) proliferative glomerulonephritis.^3^ According to the LM results in this analysis, we found endocapillary proliferative glomerulonephritis was the predominant histological pattern in typical PIGN. The least common histological pattern in adult PIGN is MsPGN (accounting for 2% of cases in one study).^26^ However, MsPGN is the most common pattern in glomerulonephritis related to staphylococcal infection. There can be focal mesangial cells and/or endocapillary proliferative or DPGN with infiltration of neutrophils and mononucleated cells within the capillary lumens using LM.^26^ DPGN and MsPGN were the predominant histological patterns observed. Varying degrees of global glomerulosclerosis, necrotizing crescentic glomerulonephritis, and cellular crescents were noted in some glomerulonephritis related to staphylococcal infection patients. These symptoms are rarely found in typical PIGN patients.

Typical PIGN is commonly characterized by glomerular granular deposition of C3 and IgG or C3 only by immunofluorescence and occasionally with IgM in PIGN.^3^ Conversely, immune-complex deposits in human glomerulonephritis related to staphylococcal infection are typically IgA-dominant or C3-dominant rather than IgG-dominant granular deposition in capillary walls detected using IF,^2,4,9^ especially in older patients with diabetes. Seldom, patients have characteristic subepithelial “hump-shaped” electron-dense deposits found in typical PIGN by EM.

Furthermore, other factors such as age of onset, gender, or underlying complications may lead to different outcomes. PIGN is generally a self-limited disease, especially in children, and treatment is mainly supportive. Compared with classic PIGN, the outcome of glomerulonephritis related to staphylococcal infection is significantly inferior. Our dataset showed remission in 44.7% of patients, which may be related to the advanced age of the patients and underlying DM.^26^

The IF features of IgA-dominant granular deposition may predispose glomerulonephritis related to staphylococcal infection to be confused with IgA nephropathy (IgAN). This former disease generally affects older men, whereas IgAN develops in young or middle-aged people.^30^ IgA and C3-predominant or codominant immune complex deposits along glomerular capillary loops and mesangial areas were detected in patients with glomerulonephritis related to staphylococcal infection, whereas IgAN showed mesangial deposits. Subepithelial “hump-like” electron-dense areas cannot be found in IgAN using EM. Other features that suggest glomerulonephritis related to staphylococcal infection and not primary IgAN include intercurrent culture-documented bacterial infection and hypocomplementemia. Treatments for these 2 disease entities are quite different. It is clear that Renin-Angiotensin System inhibitor and steroid treatment are effective in the majority of primary IgAN, but antibiotic therapy is still the first choice for glomerulonephritis related to staphylococcal infection. Thus, a correct diagnosis is critical in providing appropriate treatment for glomerulonephritis related to staphylococcal infection or IgAN.

Glomerulonephritis related to staphylococcal infection often has a more aggressive presentation, and the majority of patients present with DM, acute renal failure, hematuria, and heavy proteinuria. Previous studies indicated patients with diabetes have a high incidence of staphylococcal infection, especially of the lower-extremity skin and bone. These infections are associated with higher colonization and more frequent skin ulcerations.^26^ There are 2 studies by Nasr et al that found that 65% of adults and 55% of older patients with diabetes developed ESRD after PIGN.^3,26^ The *P* value of DM was 0.053 by the multivariate analysis, so it was reasonable to regard DM as one of the independent risk factors of ESRD or death.

Although this study included the largest number of patients with glomerulonephritis related to staphylococcal infection for pooled analysis for the first time, there might have been a number of limitations. Firstly, patients with glomerulonephritis related to staphylococcal infection were collected and pooled analyzed based on the definite diagnosis criteria in this study. But there was still some bias because of its retrospective nature. Secondly, some data were derived from published articles and were not from case reports. Thirdly, patients were diagnosed at different hospitals with probable different indications for biopsy. The ideal therapy strategy for this disease is not clear. Thus, a large number of clinical investigations are still necessary.

In conclusion, the incidence of glomerulonephritis related to staphylococcal infection has increased, particularly in the older patients with diabetes. The majority of patients are infected with MRSA. Various renal histopathologic profiles have been defined and IgA and C3-predominant or codominant findings are important features. The treatment generally involves antibiotics. Increasing age is a risk factor of poor prognosis.
